# Gut microbiome profiles and associated metabolic pathways in patients of adult-onset immunodeficiency with anti-interferon-gamma autoantibodies

**DOI:** 10.1038/s41598-022-13289-9

**Published:** 2022-06-01

**Authors:** Hui-Shan Hsieh, Yu-Nong Gong, Chih-Yu Chi, Sen-Yung Hsieh, Wei-Ting Chen, Cheng-Lung Ku, Hao-Tsai Cheng, Lyndsey Lin, Chang Mu Sung

**Affiliations:** 1 Department of Otolaryngology-Head and Neck Surgery, Xiamen Chang Gung Hospital, Xiamen, China; 2 Research Center for Emerging Viral Infections, College of Medicine, Chang Gung University, Taoyuan, Taiwan; 3 Department of Laboratory Medicine, Linkou Chang Gung Memorial Hospital, Taoyuan, Taiwan; 4 Division of Infectious Diseases, Department of Internal Medicine, China Medical University Hospital, Taichung, Taiwan; 5 School of Medicine, College of Medicine, China Medical University, Taichung, Taiwan; 6 Department of Gastroenterology and Hepatology, Chang Gung Memorial Hospital, No. 5, Fuxing St., Guishan Dist., Taoyuan, 33305 Taiwan; 7 Graduate Institute of Biomedical Science, College of Medicine, Chang Gung University, Taoyuan, Taiwan; 8 Department of Digestive Disease, Xiamen Chang Gung Hospital, Xiamen, China; 9 Laboratory of Human Immunology and Infectious Disease, Graduate Institute of Clinical Medical Sciences, Chang Gung University, Taoyuan, Taiwan; 10 Department of Nephrology, Chang Gung Memorial Hospital, Taoyuan, Taiwan; 11 Center for the Molecular and Clinical Immunology, Chang Gung University, Taoyuan, Taiwan; 12 Division of Gastroenterology and Hepatology, Department of Internal Medicine, New Taipei Municipal TuCheng Hospital, New Taipei City, Taiwan; 13 College of Medicine, Chang Gung University, Taoyuan, Taiwan; 14 Division of Internal and Pediatric Chinese Medicine, Center for Traditional Chinese Medicine, Chang Gung Memorial Hospital, Linkou, Taiwan; 15 Graduate Institute of Clinical Medicine, College of Medicine, Chang Gung University, Taoyuan, Taiwan

**Keywords:** Immunological disorders, Clinical microbiology

## Abstract

Autoantibodies against interferon-gamma (AutoAbs-IFN-γ) can cause the immunodeficiency condition following various opportunistic infections. Gut microbiota can affect the human immune system in many ways. Many studies have shown that gut dysbiosis was associated with some immune diseases, such as autoimmune diseases and human immunodeficiency virus (HIV) infection, while its relationship at anti-IFN-γ AAbs remains unknown. We aimed to identify the anti-IFN-γ AAbs specific microbiome and the possible association with immunodeficiency. We profiled fecal microbiome for two cohorts of forty subjects, including seven patients with anti-IFN-γ AAbs and 33 individuals with competent immune. The study shows that patients with anti-IFN-γ AAbs have characterized the gut microbiome and have lower alpha diversity indexes than healthy controls (HC). There are significant differences in the microbiome structure at both the family and genera level between the two cohorts. The anti-IFN-γ AAbs cohort featured some microbiome such as Clostridium, including the possible opportunistic pathogen and fewer genera including *Bacteroides*, *Ruminococcus,* and *Faecalibacterium,* some of them with possible immune-related genera*.* The PICRUSt2 pathway demonstrated the decreased abundance of some immune-related pathways and one potential pathway related to the immune alternations in the anti- IFN-γ AAbs cohort. This was the first study to examine the gut microbiome characteristics in patients with anti-IFN-γ AAbs. It could be involved in the pathogenesis of anti-IFN-γ AAbs and contribute to the derived immune condition in this disease. This could lead to new strategies for treating and preventing patients suffering from this disease.

## Introduction

Interplays between microorganisms and mammals' immune system include interplays in disease and homeostasis. The microbiome is crucial in developing important elements of the host's immunity system^1^. While the immune system organizes preservation of key aspects of the host-microbe symbiosis, The microbiome-derived toller-like receptor (TLR), and nucleotide-binding oligomerization domain (NOD) ligands, as well as metabolites such as short-chain fat acids (SCFAs), have been shown to affect enterocytes and intestinal immune cells directly. They also reach distant tissues by using systemic circulation to regulate immunity^1–3^.

Any deviate interactions between microbiome, host's immune system, and genetically susceptible individuals may lead to complex immune-mediated diseases such as inflammatory bowel disease (IBD) and systemic autoimmune diseases^4–7^. Disrupted microbiota homeostasis, also known as dysbiosis, is a potential trigger for proinflammatory conditions, pathological states-like metabolic diseases, autoimmune disorders, and HIV infection^8^.

However, selected mechanistically well-characterized microbiota-immune system interactions are depicted in some immunodeficiency disorders, like adult-onset immunodeficiency (AOID) with anti-interferon-gamma autoantibodies (anti-IFN-γ AAbs). This disease is associated with susceptibility to disseminated infections caused by opportunistic pathogens that affect only people with frail immune systems^9^. People with unclear etiology represent higher levels of anti-IFN-γ AAbs^10^ and repeated infections may lead to the autoantibodies with increased activity^11^.

The intestinal microbiota of patients with AOID who have anti-IFN-γ AAbs may different from those with normal resistance. This dysbiosis may act as a trigger and exacerbator to the immune cascade. This study will compare the microbiota diversity between patients with AOID and those with immunocompetence. We hope to uncover the microbial components might be responsible for the pathogenesis or progression of AOID, which will be helpful for future treatment and research.

## Materials and methods

### Patient and sample collection and DNA extraction

The study protocol was approved by the Research Ethics Committee China Medical University & Hospital, Taichung, Taiwan (approval No. DMR099-IRB-075(CR-9)), which waived the requirement for informed consent due to the retrospective nature of the study. All procedures were conducted following the current regulations.

From 2015 to 2016, seven patients (four males and three females, average age 68 years) met the definition of AOID with AAbs against IFN-γ^12^ were enrolled in this study. A control group of 33 normal individuals of similar age and sex was formed (Table 1). Both the AOID with anti-IFN-γ AAbs and control groups did not have any history of hospitalization within six months, no special dietary restrictions, and no antibiotics for one month. Their stools were collected with informed consent and further analyzed. Fresh fecal samples were collected and stored at -80 °C less than 1 h before DNA extraction. The experimental protocol was adapted from the Human Microbiome Project^13^. Total genomic DNA was extracted using a DNeasy PowerSoil kit (Cat #: 12888, QIAGEN, Hilden, Germany) with slight modifications. The bead solution was added to the frozen stool sample in a 15 ml Falcon tube (2.0 ml/g frozen stool; 1.8 ml/biopsy) during sample pre-processing. After vigorous vortexing the mixture for 30 s, it was incubated at 65 °C for 10 min, 95 °C for 10 min, and then incubated in a water bath for 10 min. Large particles were pelleted by centrifugation at 1500×*g* for 5 min, and 900 μl of supernatant was transferred to the PowerSoil bead tube. The manufacturer detailed the rest of the protocol, with the following exceptions about the user manual: (1) in Step 3, sample homogenization was performed using PowerLyzer^®^24 Homogenizer (Cat #: 13155, QIAGEN, Hilden, Germany) set to 4200 rpm for 45 s. (2) In Step 13, 1040 μl of Solution C4 was added. (3) In Step 16, the spin column was washed twice with 500 μl of solution C5 before elution.

**Table 1:** Clinical variables between the Anti-IFN-γ AAbs and health control groups.

	Anti-IFN-γ AAbs group (n = 7)	Health control group (n = 33)
Age	68 ± 10.1	66 ± 6.8
Gender (male/female)	4 (57.1)/3(42.9)	17 (51.5)/16 (48.5)
AST, U/L	37.1 ± 18.3	32 ± 11.2
AST, U/L	33 ± 19.1	28 ± 9.2
Creatine, mg/dL	1.1 ± 0.6	0.9 ± 0.4

Data are summarized as mean ± SD or n (%).

### Amplicon library construction for sequencing

Amplicon sequencing libraries were prepared as previously described^14^. Amplicons were visualized by running two μl of the product on 2.0% (w/v) agarose gel to confirm that a product was generated. Sample normalization was performed using SequalPrep Normalisation Plate Kit, 96-well (Cat #: A1051001, ThermoFisher, Waltham, Massachusetts, U.S.). Normalized amplicon products were pooled at equal volumes. The pooled DNA library was concentrated using an equal volume of Agencourt AMPure XP beads (Cat #: A63880, Beckman Coulter, Pasadena, California, U.S.). The NGS High Throughput Genomics Core performed sequencing in Biodiversity Research Centre, Academia Sinica, Taiwan. Sequencing of the 16S amplicon was carried out using Illumina MiSeq with paired-end 2 × 250 bp chemistry.

### Sequencing data processing pipeline and statistical analysis

Raw fastq files were first pre-processed using the Moving Pictures tutorial from QIIME 2(v2021.4.0.21)^15^, and taxonomically classified using the Greengenes databank (v13.8)^16^. Forward and reverse read sequences in 250-bp length were trimmed off after positions 219 and 198, respectively. Then, the operational taxonomic unit (OTU) table was imported and analyzed by a Phyloseq package (v1.28.0)^17^ in the R environment (v3.6.1). Statistical tests for health control (n = 33) and disease (n = 7) groups in alpha diversity were estimated using the Wilcoxon test. Alpha diversity rarefaction analysis was performed and visualized by Qiime2 and Qiime2 view websites, respectively. The dissimilarity indices between samples were calculated using the vegdist function with the Jaccard method, which was further ordinated using principal coordinate analysis (PCoA) in the Vegan package (v2.5–7, https://cran.r-project.org/web/packages/vegan/index.html). Furthermore, the linear discriminant analysis effect size (LEfSe)^18^ was performed for comparing their microbiome communities and cladograms. The input file for this LEfSe analysis was generated using a Dokdo Python package (https://github.com/sbslee/dokdo). Finally, the pipeline of Phylogenetic Investigation of Communities by Reconstruction of Unobserved States (PICRUSt2)^19^ in the QIIME2 plugin was performed for predicting functional abundances and the KEGG pathway. Statistical analysis of taxonomic and functional profiles (STAMP)^20^ was further used for conducting statistical tests and visualizing the profile of feature abundance generated by PICRUSt2.

## Results

### Dysbiosis of microbiome diversity in adult-onset immune deficiency (AOID) patients secondary to anti-IFN-γ AAbs

**Figure 1:**
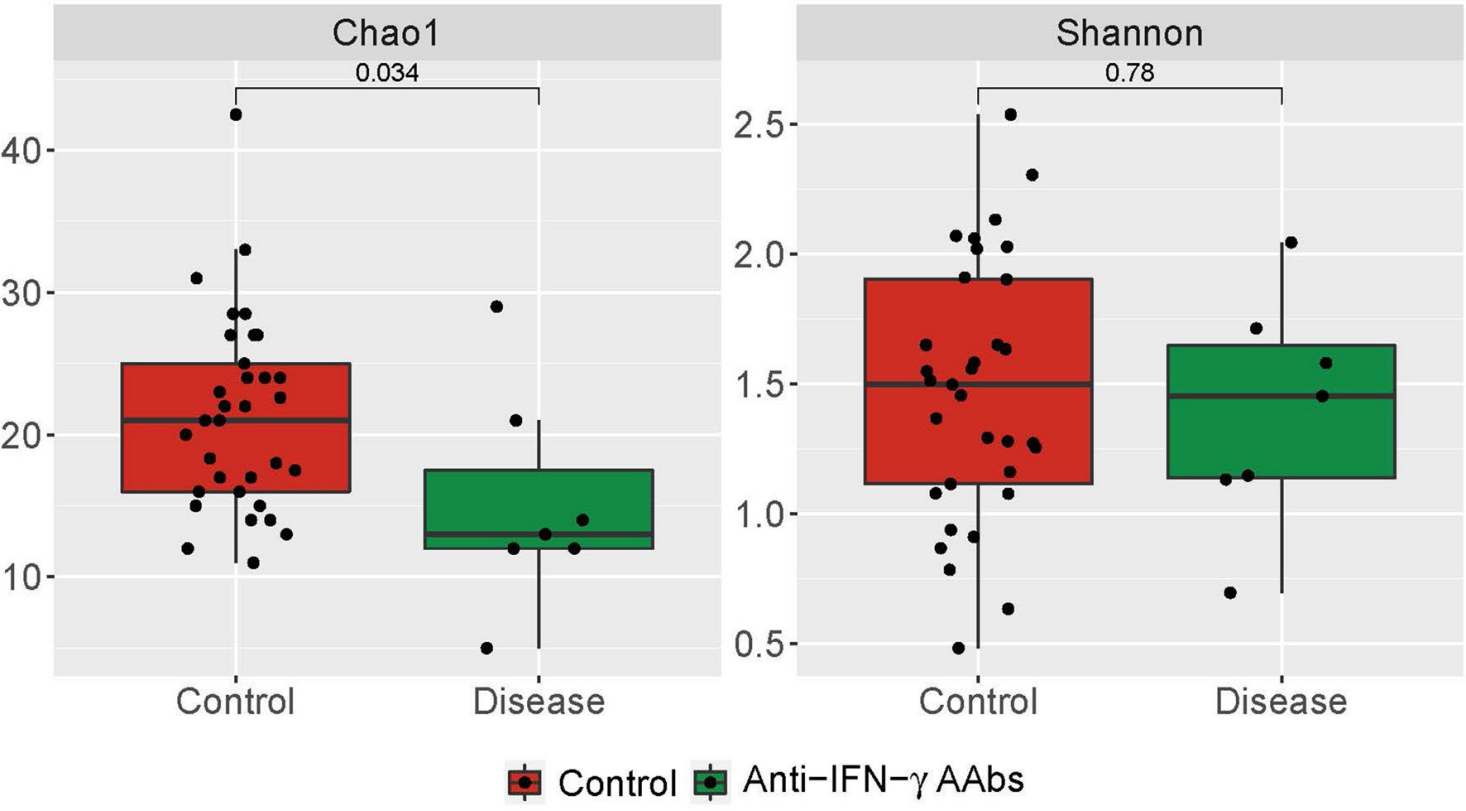
Alpha diversity of anti-IFN-γ AAbs and HC groups. The comparison of gut microbiota alpha diversity between anti-IFN-γ AAbs and HC samples, including species richness (represented by Chao 1 index) and evenness (represented by Shannon index). The Chao1 (left) and Shannon (right) diversity between anti-IFN-γ AAbs and HC were calculated using a rarefied OTU matrix. Chao diversity index in Anti-IFN-γ AAbs is significantly lower than HC, whereas the Shannon diversity showed a similar tendency without significance compared to HC. *anti-IFN-γ AAbs* anti-interferon-gamma autoantibodies, *HC* healthy control, *OTU* operational taxonomic unit.

The alpha diversity of microbiota communities was determined by two ecological parameters: Chao1 richness (a measure of OTU presence/absence) and Shannon diversity (a measure of OTU abundance/absence) in Fig. 1. The Chao1 index (OTU presence/absence) was lower than the healthy control (HC). This indicates that anti-IFN-γ AAbs may have contributed to the significantly decreased species richness (HSD; adjusted p = 0.03). The Shannon diversity index (OTU counts weighted by abundance), which measures the diversity of microbial species in a community^21^, shows a non-significant decline (HSD; adjusted = 0.78) in anti-IFN-γ AAbs patients compared to HC. This could be caused by a decrease in rare taxa but not a significant change in their abundance.

**Figure 2:**
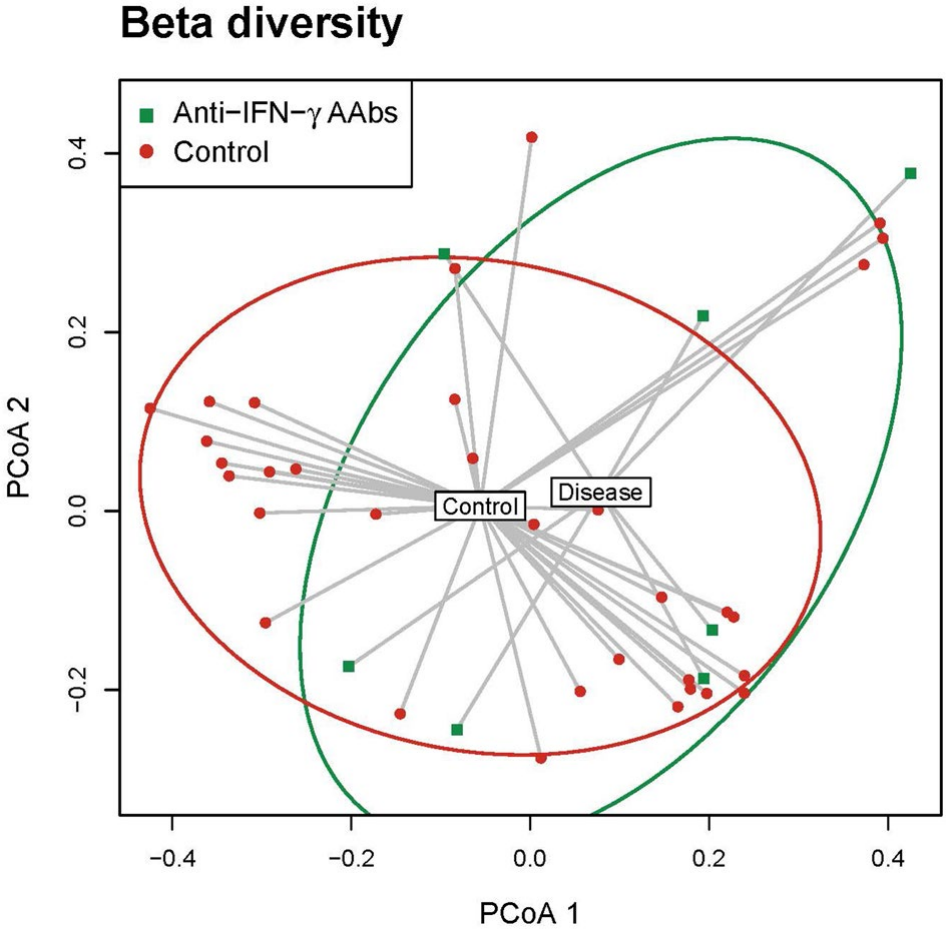
Beta diversity of anti-IFN-γ AAbs and HC groups. PCoA plot based on Jaccard dissimilarity of Anti-IFN-γ AAbs and HC samples using rarefied OTU matxis. Each symbol represents a sample, and the corresponding group can be found in the legend. Distances between any pair of samples represent the dissimilarities between each sample. *anti-IFN-γ AAbs* anti-interferon-gamma autoantibodies, *HC* healthy control, *OTU* operational taxonomic unit, *PCoA* principal coordinates analysis.

Furthermore, Beta analyses to calculate distances between the fecal samples from the HC and anti-IFN-γ AAbs groups. The overall profile of the microbial composition between HC and anti-IFN-γ AAbs patients was generated by PCoA, based on the relative difference abundance of bacterial taxa for visualization of whether the two groups have different microbial communities. As shown in Fig. 2, there was a distinct but insignificant (*p* = 0.7) clustering pattern of HC compared to the anti-IFN-γ AAbs patients. The anti-IFN-γ AAbs group mainly clustered together at the right part and shifted to the right of HC and more vertically to HC. The insignificancy might be given the rarity of anti-IFN-γ AAbs cases with the limited sample size^12^.

### Characteristics of the fecal microbiome in anti-IFN-γ AAbs patients at the phylum level

**Figure 3:**
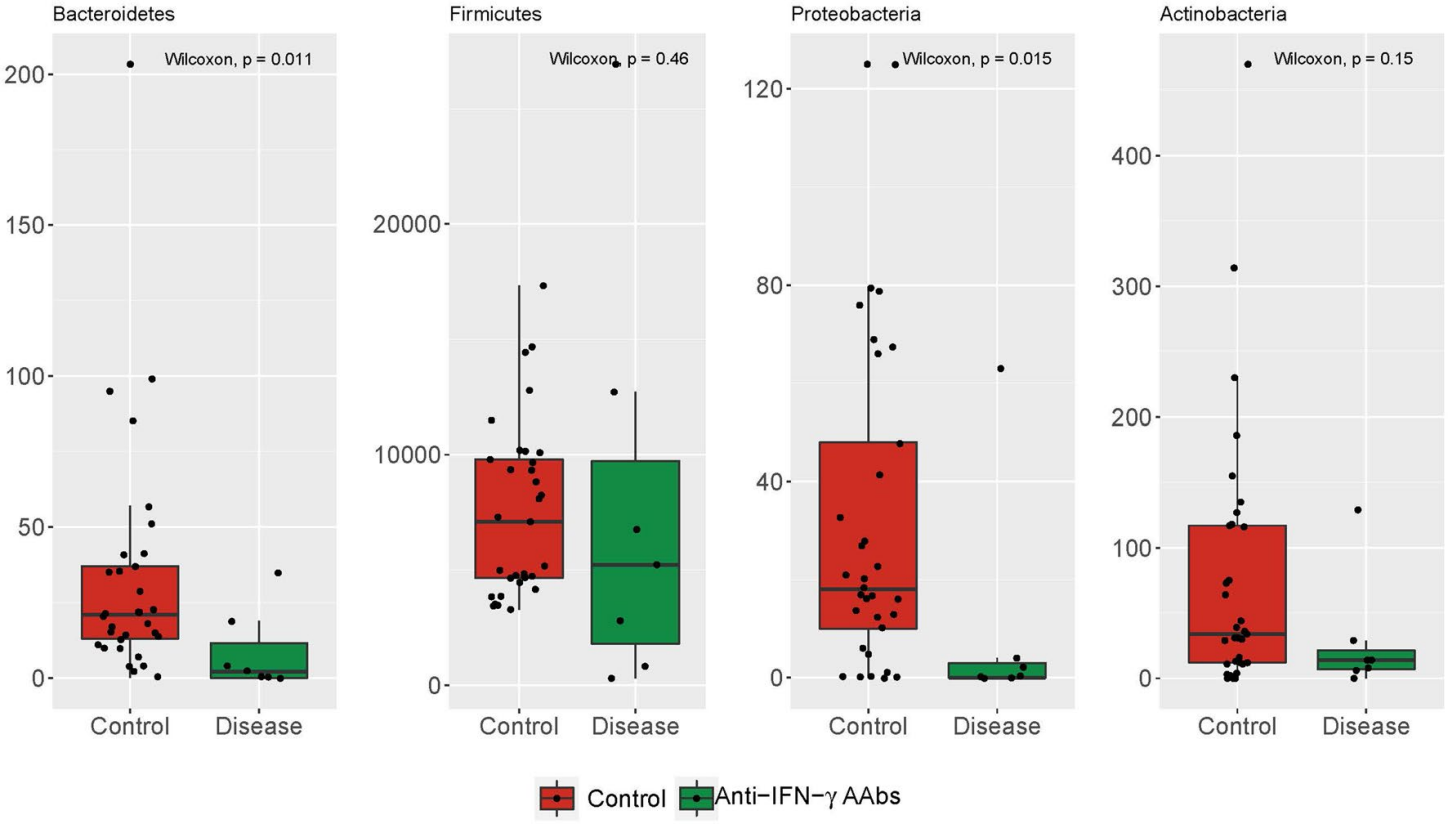
The relative abundances of the 4 major phyla of the fecal samples between Anti-IFN-γ AAbs and HC cohorts. Boxplots show the interquartile ranges and medium relative abundances. *anti-IFN-γ AAbs* anti-interferon-gamma autoantibodies, *HC* healthy control.

To demonstrate whether the anti-IFN-γ AAbs also alternated gut microbiota pattern, the proportions of 16S rRNA reads assigned to each phylum were analyzed. Fecal samples from the anti-IFN-γ AAbs group and HC were dominated by *Firmicutes, Bacteroidetes, Proteobacteria, and Actinobacteria* (99% of all phylum). In anti-IFN-γ AAbs patients, the microbial composition was characterized by the relative abundance of each taxon, including *Bacteroidetes*, *Firmicutes*, *Proteobacteria*, and *Actinobacteria*. The relative abundance of these phyla was further scrutinized amongst the controls and anti-IFN-γ AAbs patients (Fig. 3). The anti-IFN-γ AAbs patients had a significantly different fecal microbiome relative abundance to the HC. This included significantly decreased Bacteroides, and Proteobacteria (Wilcoxon, p = 0.01 and 0.02, respectively). In contrast, the *Firmicutes* and *Actinobacteria* showed an insignificant decrease*.* These diminished proportions of these four primary phyla indicate a less diverse microbiota in the anti-IFN-γ AAbs patients, consistent with the finding of declines in Chao richness and Shannon diversity.

**Figure 4:**
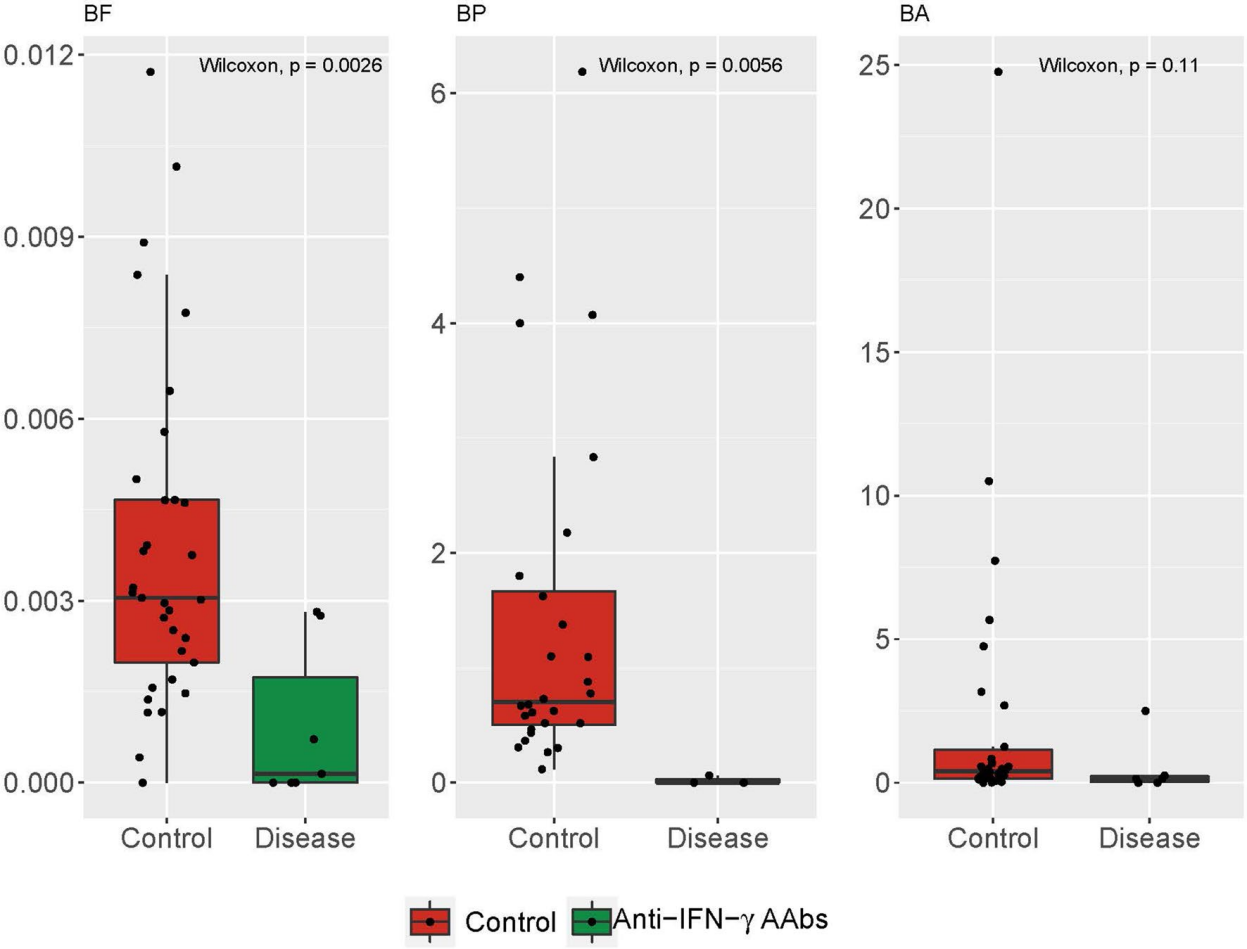
The relative abundances of the 4 major phyla of the fecal samples between Anti-IFN-γ AAbs and HC cohorts. Different combinations of Bacteroidetes/phyla between HC and Anti-IFN-γ AAbs groups. *anti-IFN-γ AAbs* anti-interferon-gamma autoantibodies, *HC* healthy control.

Moreover, the ratio between *Firmicutes*/*Bacteroidetes* (*F*/*B*) has been associated with host homeostasis maintenance^22^. Alternation in this ratio has been widely recognized as a sign of gut dysbiosis^23^. This could lead to many pathologies such as obesity, hypertension and acute hepatic encephalopathy ^23–25^. Accordingly, to survey if the ratios between *Bacteroidetes* combinations of the other three major phyla (*Firmicutes, Proteobacteria, and Actinobacteria*) can be used to differentiate the anti-IFN-γ AAbs group against HC (Fig. 4), we found the ratios of B/F and B/P were both significantly lower in anti-IFN-γ AAbs group compared to HC (both Wilcoxon, *p* < 0.01). HC had a lower B/A ratio than the anti-IFN g AAbs group, while there was no significant difference in B/A between them.

### A significant difference in the comparison of microbiota profile between anti-IFN-γ AAbs patients and HC

**Figure 5:**
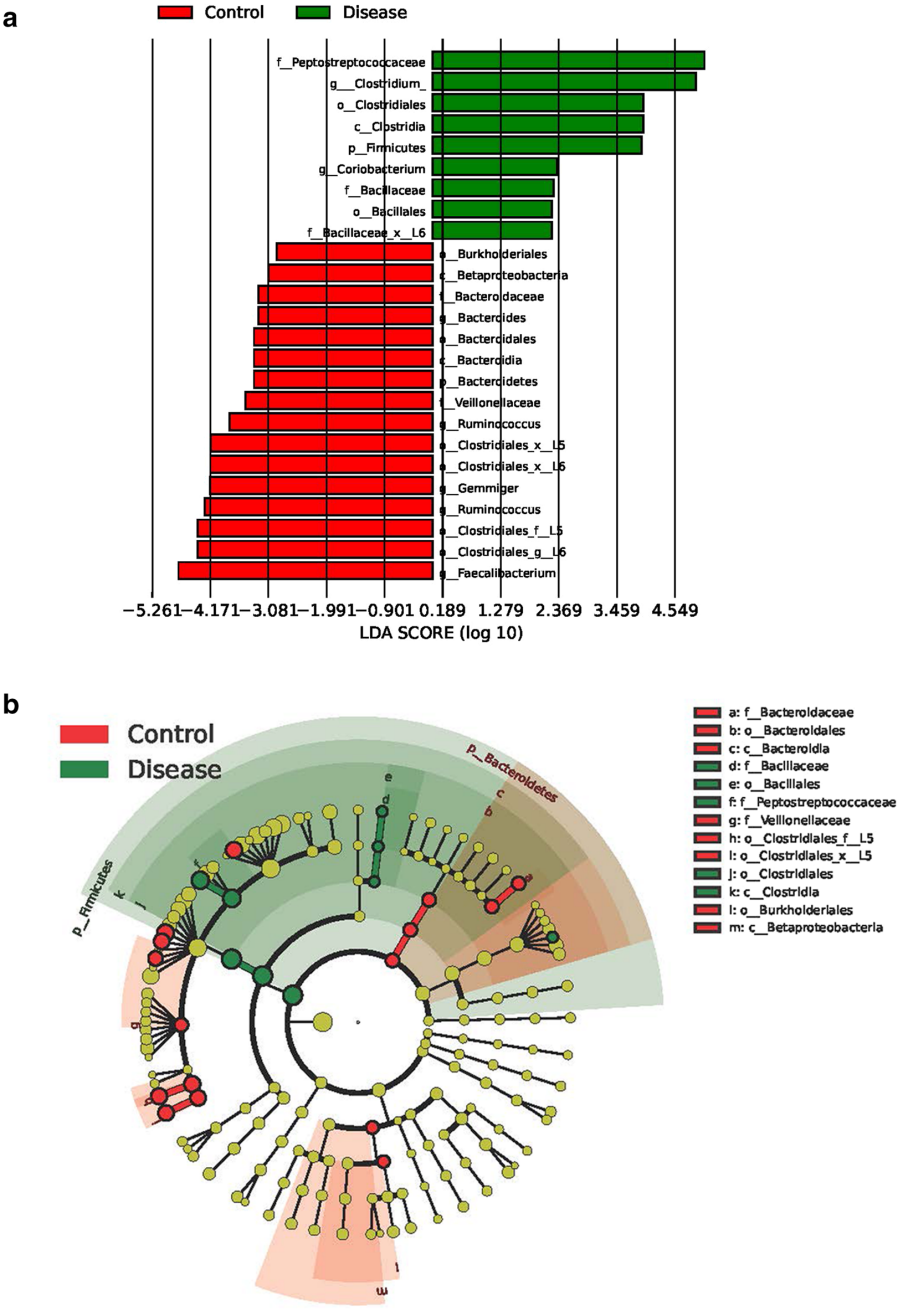
Taxonomic differences and inferred functional content of gut microbiota of HC and Anti-IFN-γ AAbs cohorts. LefSe results for the bacterial taxa were significantly different between Anti-IFN-γ AAbs and HC groups. (**A**) LDA scores show significant differences between groups. Taxa with LDA score (log 10) > 2.4 are shown, and (**B**) the cladogram showing differentially abundant taxonomic clades. The size of the node represents the abundance of taxa. From phylum to genus, each node represents a taxonomic level. Red nodes demonstrate enriched taxa in the HC group; green nodes represent enriched taxa in the anti-IFN-γ AAbs group. *anti-IFN-γ AAbs* anti-interferon-gamma autoantibodies, *HC* healthy control, *LEfSe* LDA effect size.

The LefSe algorithm was used to identify the differentially abundant microbiome in anti-IFN-γ AAbs people. It allowed for the comparison of gut microbiota of patients with anti-IFN-γ AAbs and HC. This revealed significant bacterial discrimination abundance taxonomic clades with LDA scores > 2.4 between the anti-IFN-γ AAbs group and HC (Fig. 5A,B). The genus *Bacteroides* (phylum *Bacteroidetes*)*, Ruminococcus* (phylum *Firmicutes*)*, Gemmiger* (phylum *Firmicutes*), and *Faecalibacterium* (phylum *Firmicutes*) were the main dominant genera enriched in HC. Faecalibacterium, a taxon related to colon epithelium repairs and Treg cell production was significantly lower in patients with anti-IFN-γ AAbs than the HC^26^. We also saw a decrease in SCFAs-producing bacteria, including *Bacteroides, Ruminococcus, and Faecalibacterium*, along with an enrichment of *Clostridium* (phylum *Firmicutes*) and *Coriobacterium* (phylum *Actinobacteria*), among anti-IFN-γ AAbs individuals. Notably, *Clostridium* is the taxon that include many opportunistic, pathogenic species. They were enriched in anti-IFN-γ AAbs patients compared to the HC.

### Functional prediction of PICRUSt2 analysis associated with anti-IFN-γ autoantibodies patients

**Figure 6:**
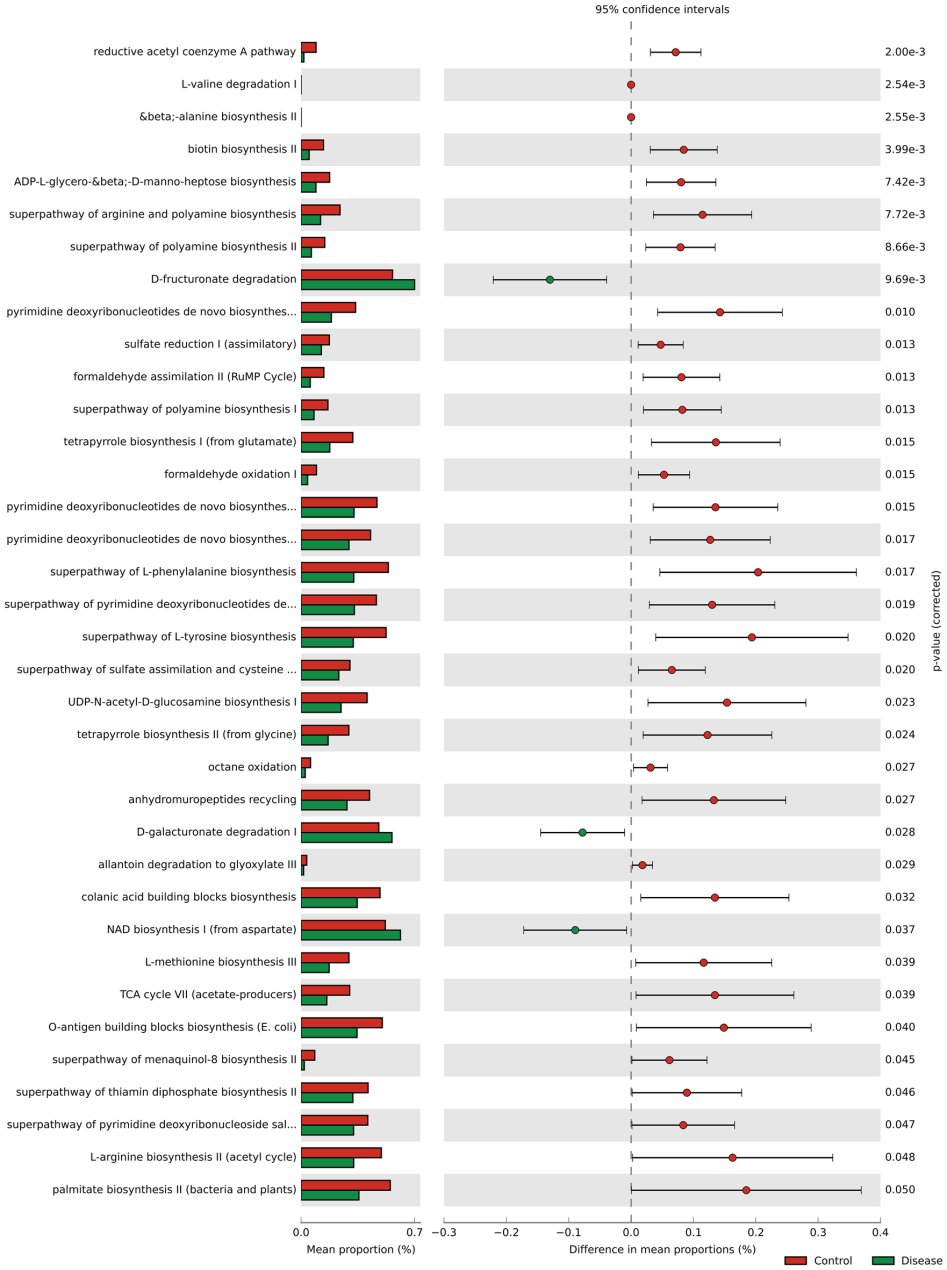
Predicted differential KEGG pathways in HC and Anti-IFN-γ AAbs. The extended error bar plot of significantly differential KEGG pathways was predicted using PICRUSt2 analysis. Bar plots on the left side display the mean proportion of each KEGG pathway. Dot plots on the right show the differences in mean proportions between the two indicated groups using *p* value. Only *p* value < 0.05 (Welch’s test) are shown. *anti-IFN-γ AAbs* anti-interferon-gamma autoantibodies, *HC* healthy control.

The PICRUSt2 analysis predicted the gut microbial functional pathways to determine if the taxonomic variations correspond to potential functional changes (Fig. 6). There are 31 pathways that have been significantly enriched with HC. These include pathways related to butyrate (reductive acetyl-coenzyme A pathway), polyamine biosynthesis pathways (superpathway of arginine, polyamine biosynthesis and suprapathway of polyamine synthesis), which could transport putrescine or spermidine. These pathways can modulate immunity and reduce inflammation in the gut^27^. Three PICRUSt2 pathways are significantly higher in individuals with anti-IFN-γ autoantibodies (Welch’s t-test; p 0.05), and these include d-fructuronate degradation, d-galacturonate degradation I, as well as NAD biosynthesis I (from spermidine).

## Discussion

Numerous reports have been published on the importance of host immune-microbiota interactions for maintaining and developing homeostasis in the human organism^28–30^. Additionally, HIV infection^31,32^ was linked to other autoimmune diseases such as Type I diabetes (T1D)^33,34^, and Behcet's disease (BD)^35,36^. HIV encourages replication by T-helper lymphocytes and the relationship between retrovirus infection, gut dysbiosis and chronic systemic inflammation are linked^37^. T1D is a chronic, systemic disease that causes autoimmunity to destroy beta cells. A combination of dysbiosis and impaired intestinal immune response may lead to anti-islet autoimmunity^38^. BD, the vasculitis disease with a possible association between specific gut microbiome alternations and defects in T helper cells and regulatory T cells (Treg cells)^39^. There is little information about the effects of the microbiota in adult-onset immunodeficiency (AOID), which was first described in 2004–2005 in patients with no medical history and who had developed a severe or disseminated infection from environmental non-tuberculous mycobacteria^40–42^.

In this study, we used the fecal samples for 16S rRNA sequencing analysis to characterize and compare the gut microbiome profiles between the anti-IFN-γ AAbs patients and healthy individuals, which was from a different aspect of the previous AOID-related pathologic molecular genetic report^43^. Notably, we revealed that anti-IFN-γ autoantibodies harbored distinct microbiota in their guts compared with healthy control. The average number of reads was significantly lower in anti-IFN-γ AAbs patients, indicating a reduced species richness. Moreover, the result further implies a decrease in rare taxa but not a significant change in their abundance. We also found that the cluster analysis at the genus level revealed not significant but two distinct clusters from the anti-IFN-γ AAbs group and HC, further implying different compositions between these two groups. Moreover, we believe that providing a detailed profile of fecal microbiomes of anti-IFN-γ AAbs patients allows a possible non-invasive identification method and treatment of this entity.

Our study revealed that the anti-IFN-γ AAbs group decreased alpha diversity compared to healthy controls. This could result from altered gut microbiomes in anti-IFN-γ AAbs patients, weakening the intestinal epithelium barrier and leading to opportunistic infections and complications. The fecal microbial community with diminished proportions of four primary phyla, including a significant decrease of the phylum *Bacteroidetes* and *Proteobacteria*, accompanied with reduced abundance of the phylum *Firmicutes* and *Actinobacteria*, indicate insignificant decrease biodiversity of microbiota in the anti-IFN-γ AAbs patients, which were consistent with the decreases in Chao richness and Shannon diversity. We found that the predominant bacteria in patients with anti-IFN-γ AAbs were acetate-producing bacteria such as *Bacteroides* and some butyrate-producing bacteria such as *Ruminococcus* or *Faecalibacterium*. These genera can metabolize various carbohydrate substrates to create SCFAs, including acetate, propionate, and butyrate^44^. This decrease in fermentation-related bacteria decreases SCFA production, a preferred substrate for energy for colonocytes. It also promotes cell reparation, and differentiation protects the integrity of intestinal barrier function^45–47^ and that decline in anti-IFN-γ AAbs individuals. SCFAs have been reported to promote the development of some autoimmune diseases, enhance interleukin-10 (IL-10) release, activate Treg cells, inhibit the production of proinflammatory cytokines, such as NF-kB, and alleviate colonic inflammation^47,48^. Anti-IFN-γ AAbs-associated dysbiosis has been described as an imbalance in specific microbial populations, their corresponding metabolic metabolites, and impaired induction or activation of Treg cells. This may also lead to reduced SCFAs-producing bacteria, which may cause impaired induction of Tregs and further expose individuals to lower anti-inflammatory activity. It may also provide a potential pathway for pathogen translocation. The investigation of PICRUSt2 analysis in sequenced samples in predicting pathways among anti-IFN-γ AAbs groups and HC was consistent with the observation of the decreases in butyrate-producing bacterias among anti-IFN-γ AAbs patients. The acetyl-CoA pathway, commonly referred to as the Wood-Ljungdahl pathway or the reductive acetyl-CoA pathway, is one of the major metabolic pathways utilized by bacteria^49^. This pathway is one of the butyrate-producing pathways^50^ used by acetate-producing bacteria, also displayed a downward tendency in anti-IFN-γ AAbs individuals (*p* = 1.99e−03). This downregulation implied lower SCFAs production in anti-IFN-γ AAbs individuals and was consistent with the previous gut-microbiome findings in our study.

Another notable observation was that two identified pathways involved in polyamine biosynthesis (superpathway of arginine and polyamine biosynthesis, and superpathway of polyamine biosynthesis) significantly decreased in the gut of anti-IFN-γ AAbs individuals compared to HC. Systematic metabolomic analysis has clearly shown that the gut microbiota can both produce polyamines (i.e., putrescine, spermidine, and spermine) and mammalian cells; therefore, they have been considered co-metabolites as the compounds derived from the interaction between microbiota and host metabolism^51^. Their significant functions in the gut are the intestinal epithelial barrier, as well as the modulation of immunity and anti-inflammatory effects, that evidenced from the conversion of intraepithelial CD4^+^ T helper cells into Treg lymphocytes and inhibition of Th17 cell polarization in mice^33^, and spermidine was reported as a protective role in an animal model of autoimmune diseases of multiple sclerosis and psoriasis^52,53^. Furthermore, intestinal alterations in polyamine production have also been observed in circulation and associated with graft-versus-host disease^54^. In our study, the pathways related to polyamines biosynthesis depleted in anti-IFN-γ AAbs patients compared to HC is intriguing to speculate the alternation of this co-metabolites might also contribute to triggering and developing the immune system of anti-IFN-γ AAbs individuals. Notwithstanding, further investigations are warranted to clarify the complex functional interconnections; this alternation in our report might indicate a possible effective area of future drug targeting for anti-IFN-γ AAbs patients.

In addition to the pathways mentioned above, the NAD biosynthesis pathway upregulated in anti-IFN-γ AAbs patients is also noted. Extracellular NAD+ restores intracellular NAD+ pools and plays a significant part in immune modulation. Inflammation can trigger intracellular NAD+ releasing into the extracellular space, which conversely fine-tunes the immune response^55^. In other words, extracellular NAD+ serves as a proinflammatory cytokine that stimulates granulocytes to augmentin chemotaxis^56^. This finding is consistent with the report of immune alternation with increased monocytes in anti-IFN-γ AAbs patients^57^. This NAD biosynthesis of anti-IFN-γ AAbs patients increases the proinflammatory status that may cascade the following autoantibodies production and immunocompromised status.

## Conclusions

In sum, our results revealed that specific bacterial taxa are associated with the anti-IFN-γ AAbs. This is the first study that comparatively evaluated gut microbiota features in anti-IFN-γ AAbs, providing new insight into the association and possible links between gut microbiota dysbiosis and anti-IFN-γ AAbs. Based on our findings above, a reduced abundance of butyrate-producing and acetate-producing bacteria may lead to depression in SCFAs, which may be the basic physiological for low-level inflammation of anti-IFN-γ AAbs. Further exploration may reveal they act as probiotics as a novel treatment for AOID. As a result of depletion of these beneficial bacteria tipped the host immune homeostasis and led to overgrowth of some opportunistic pathogens to cause initial infection and succeeding immune alternations. Meanwhile, the PICRUSt2 pathways analysis indicates decreased abundance of some possible metabolism-related pathways in anti-IFN-γ AAbs individuals, which might associate gut dysbiosis with dysregulation of the metabolic and immunologic functions contributing to the anti-IFN-γ AAbs development. These taxa help distinguish individuals with anti-IFN-γ AAbs from healthy controls. Despite we cannot rule out the possibility that the rare scenario of anti-IFN-γ AAbs with a limited sample size and their therapeutic medication may have the potential influence on these results, our findings indicate that the roles of the gut microbiota and the possible specific mechanisms in anti-IFN-γ AAbs patients that provide the direction are worth to be further investigated and validated with larger scaled cohort and metabolic analyses.

## Data Availability

The data of this study are available from the corresponding author upon reasonable request. Y.N.G. and C.M.S. have full access to all the data in the study. They take responsibility for the integrity of the data, the accuracy of the data analysis, and the conduct of the research.
